# Alpha-synuclein aggregation induces prominent cellular lipid changes as revealed by Raman spectroscopy and machine learning analysis

**DOI:** 10.1093/braincomms/fcaf133

**Published:** 2025-04-03

**Authors:** Nathan P Coles, Suzan Elsheikh, Agathe Quesnel, Lucy Butler, Ojodomo Achadu, Meez Islam, Karunakaran Kalesh, Annalisa Occhipinti, Claudio Angione, Jon Marles-Wright, David J Koss, Alan J Thomas, Tiago F Outeiro, Panagiota S Filippou, Ahmad A Khundakar

**Affiliations:** School of Health & Life Sciences, Teesside University, Middlesbrough TS1 3BX, UK; National Horizons Centre, Teesside University, Darlington DL1 1HG, UK; School of Health & Life Sciences, Teesside University, Middlesbrough TS1 3BX, UK; National Horizons Centre, Teesside University, Darlington DL1 1HG, UK; School of Health & Life Sciences, Teesside University, Middlesbrough TS1 3BX, UK; National Horizons Centre, Teesside University, Darlington DL1 1HG, UK; School of Health & Life Sciences, Teesside University, Middlesbrough TS1 3BX, UK; National Horizons Centre, Teesside University, Darlington DL1 1HG, UK; School of Health & Life Sciences, Teesside University, Middlesbrough TS1 3BX, UK; National Horizons Centre, Teesside University, Darlington DL1 1HG, UK; School of Health & Life Sciences, Teesside University, Middlesbrough TS1 3BX, UK; National Horizons Centre, Teesside University, Darlington DL1 1HG, UK; School of Health & Life Sciences, Teesside University, Middlesbrough TS1 3BX, UK; National Horizons Centre, Teesside University, Darlington DL1 1HG, UK; National Horizons Centre, Teesside University, Darlington DL1 1HG, UK; School of Computing, Engineering & Digital Technologies, Teesside University, Middlesbrough TS1 3BX, UK; Centre for Digital Innovation, Teesside University, Middlesbrough TS1 3BX, UK; National Horizons Centre, Teesside University, Darlington DL1 1HG, UK; School of Computing, Engineering & Digital Technologies, Teesside University, Middlesbrough TS1 3BX, UK; Centre for Digital Innovation, Teesside University, Middlesbrough TS1 3BX, UK; Biosciences Institute, Cookson Building, Framlington Place, Newcastle University, Newcastle upon Tyne NE2 4HH, UK; Division of Neuroscience, School of Medicine, University of Dundee, Nethergate, Dundee DD1 4HN, Scotland; Translational and Clinical Research Institute, Faculty of Medical Sciences, Newcastle University, Newcastle upon Tyne NE2 4HH, UK; Translational and Clinical Research Institute, Faculty of Medical Sciences, Newcastle University, Newcastle upon Tyne NE2 4HH, UK; University Medical Center Göttingen, Department of Experimental Neurodegeneration, Center for Biostructural Imaging of Neurodegeneration, Von-Siebold-Straße 3a, 37075 Göttingen, Germany; Max Planck Institute for Multidisciplinary Sciences, Am Faßberg 11, 37077 Göttingen, Germany; Deutsches Zentrum für Neurodegenerative Erkrankungen (DZNE), Von-Siebold-Straße 3A, 37075 Göttingen, Germany; School of Health & Life Sciences, Teesside University, Middlesbrough TS1 3BX, UK; National Horizons Centre, Teesside University, Darlington DL1 1HG, UK; Laboratory of Biological Chemistry, School of Medicine, Faculty of Health Sciences, Aristotle University of Thessaloniki, Thessaloniki 54124, Greece; School of Health & Life Sciences, Teesside University, Middlesbrough TS1 3BX, UK; National Horizons Centre, Teesside University, Darlington DL1 1HG, UK; Translational and Clinical Research Institute, Faculty of Medical Sciences, Newcastle University, Newcastle upon Tyne NE2 4HH, UK

**Keywords:** α-synuclein aggregation, Raman spectroscopy, lipids, neurodegeneration, machine learning

## Abstract

The aggregation of α-synuclein is a central neuropathological hallmark in neurodegenerative disorders known as Lewy body diseases, including Parkinson's disease and dementia with Lewy bodies. In the aggregation process, α-synuclein transitions from its native disordered/α-helical form to a β-sheet-rich structure, forming oligomers and protofibrils that accumulate into Lewy bodies, in a process that is thought to underlie neurodegeneration. Lipids are thought to play a critical role in this process by facilitating α-synuclein aggregation and contributing to cell toxicity, possibly through ceramide production. This study aimed to investigate biochemical changes associated with α-synuclein aggregation, focusing on lipid changes, using Raman spectroscopy coupled with machine learning. HEK293, Neuro2a and SH-SY5Y expressing increased levels of α-synuclein were treated with sonicated α-synuclein pre-formed fibrils, to model seeded aggregation. Raman spectroscopy, complemented by an in-house lipid spectral library, was used to monitor the aggregation process and its effects on cellular viability over 14 days. We detected α-synuclein aggregation by assessing β-sheet peaks at 1045 cm⁻^1^, in cells treated with α-synuclein pre-formed fibrils, using machine learning (principal component analysis and uniform manifold approximation and projection) analysis based on Raman spectral features. Changes in lipid profiles, and especially sphingolipids, including a decrease in sphingomyelin and increase in ceramides, were observed, consistent with oxidative stress and apoptosis. Altogether, our study informs on biochemical alterations that can be considered for the design of therapeutic strategies for Parkinson's disease and related synucleinopathies.

## Teaser

α-Synuclein aggregation, central to neurodegenerative disorders like Parkinson’s disease and dementia with Lewy bodies, involves transitions to β-sheet-rich structures forming aggregates. Coles *et al.* used Raman spectroscopy and machine learning to analyse α-synuclein aggregation and lipid alterations, revealing reductions in sphingomyelin and increases in ceramide, consistent with oxidative stress and apoptosis.

## Graphical Abstract

**Graphical Abstract:**
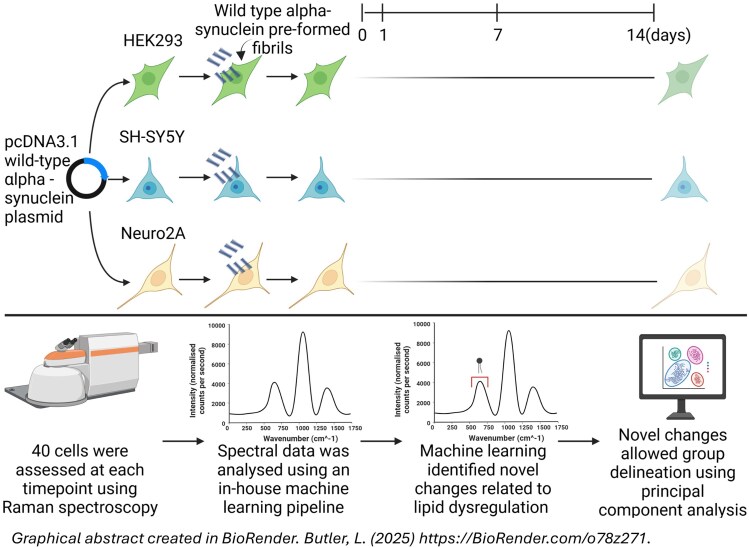

## Introduction

The aggregation of α-synuclein is a key pathological hallmark in Lewy body diseases, including Parkinson's disease and dementia with Lewy bodies (DLB).^1-4^ α-Synuclein is thought to aggregate from its native intrinsically disordered monomeric form, undergoing a profound structural transition through an α-helical structure to a β-sheet-rich filamentous configuration.^5,6^ This process leads to the formation of toxic oligomers and protofibrils and fibrils, which ultimately mature to form neuronal cytoplasmic inclusions termed ‘Lewy bodies’.^7^

Lipids are thought to play a crucial role in α-synuclein aggregation and the formation of Lewy bodies and have been shown to co-aggregate with misfolded α-synuclein.^8,9^ This process involves alterations in membrane properties and composition, promoting α-synuclein aggregation into amyloid fibrils.^10,11^ α-Synuclein possesses affinity for lipid membranes linked to its structure, with its N-terminal region (aa 1–60) containing amphipathic α-helix motifs and allowing the formation of electrostatic interactions with negatively-charged acidic membrane lipids.^12^ The central hydrophobic domain (aa 61–95), responsible for β-sheet formation, promotes aggregation.^13^ The C-terminal region (aa 96–140) is negatively charged and disordered but may interact with membranes in the presence of Ca^2+^.^14^ Such structural features, combined with membrane properties like hydrophobicity and charge, modulate α-synuclein membrane-binding. Dysregulated lipid metabolism, particularly involving sphingomyelin and phosphatidylcholine, is thought to contribute to neurotoxicity by promoting the production of ceramide,^15,16^ which induces apoptosis through increased reactive oxygen species.^17,18^ Analysis of post-mortem Lewy bodies from Parkinson's disease cases has shown heightened glucocerebrosidase activity and lipid accumulation,^19,20^ including the presence of sphingomyelin and phosphatidylcholine^20^ and phosphoinositide,^21^ likely resulting from membrane disruption caused by pathological α-synuclein. Altered ceramide metabolism is also thought to be a factor in the extracellular vesicle-mediated spread of α-synuclein in Lewy body disorders.^22^

To study α-synuclein aggregation *in vitro*, several cellular models have been created to replicate this pathological process.^23-26^ These models have unveiled notable biochemical changes linked to α-synuclein overexpression, which mimic familial forms of Parkinson's disease associated with SNCA gene multiplications. They also offer valuable insights into the pathological impacts on cellular viability and function. For example, the incubation of primary neuronal cultures with sonicated α-synuclein aggregates has shown elevated autophagy markers by Day 10, and cell death and mitochondrial stress by Day 14.^25^ Overexpression of wild-type α-synuclein has been shown to impair macroautophagy in SKNSH human neuroblastoma cells, through mitochondrial disruption and inhibition of Rab1a, a regulator of ER-Golgi trafficking.^27^ Similarly, in SH-SY5Y cells, high molecular weight (HMW) α-synuclein oligomers have been shown to disrupt cellular membranes, triggering caspase-dependent apoptosis.^18^ Other detrimental effects of α-synuclein aggregation, such as membrane disruption,^18,28^ mitochondrial dysfunction,^29^ loss of neuronal excitability,^26^ disruption of axonal transport^24^ and dysregulation of Ca^2+^ homeostasis resulting in autophagy^30^ have been found. α-Synuclein aggregation has also been found to upregulate acidic sphingomyelinase activity, converting sphingomyelin into cerebrosides and subsequently ceramide, which triggers apoptosis by increasing reactive oxygen species production.^17,18,31^

Recently, Raman spectroscopy has emerged as a powerful tool for cellular analysis, providing a rapid and non-destructive method that does not require sample pre-treatment, thus making it highly efficient for studying cellular processes.^32^ Our group has recently demonstrated that confocal Raman microscopy combined with machine learning can effectively discriminate between low and/or high-grade glioma samples in single-cell and spheroid models.^33^ Raman spectroscopy and machine learning have also identified pathophysiological changes in various cancers, including liver,^34^ breast,^35^ prostate^36^ and gastric^37^ cancer. This technique provides detailed biochemical information, revealing novel biomolecular changes related to cellular health and processes. For instance, in gastric carcinoma cells, decreases at 782 cm⁻^1^, related to nucleic acid bases, suggest changes in cellular autophagy,^38^ while in proliferative human hepatocytes, attenuated bands at 1003 cm⁻^1^ (reactive oxygen species), 1206 cm⁻^1^ (hydroxyproline) and 1440 cm⁻^1^ (triglyceride) indicate alterations during cellular differentiation.^39^ Raman spectroscopy is particularly effective for lipid analysis due to their non-polar nature, which provides a strong Raman scattering cross-section. This is driven by the highly polarizable C–H and C–C bonds in their hydrocarbon chains, making the technique especially sensitive to lipid structures.^40^ This sensitivity to molecular vibrations makes Raman ideal for investigating lipid-rich environments, such as those involved in α-synuclein aggregation.^40^

In this study, we employed a novel workflow combining Raman spectroscopy with machine learning techniques to investigate the molecular mechanisms underlying α-synuclein aggregation, with a specific focus on lipid molecule changes. We applied this method to evaluate α-synuclein aggregation in cultured cell lines overexpressing α-synuclein and treated with pre-formed fibrils (PFFs) of recombinant wild-type (WT) α-synuclein. We indirectly assessed the aggregation process and its impact on cellular viability by monitoring biochemical changes over 14 days using Raman spectroscopy, followed by machine learning techniques to reduce data dimensionality, allowing assessment of delineation between treated and non-treated cells. To support the analysis of neurolipid biomolecules involved in Lewy body formation, an in-house spectral library of lipid standards, including phospholipids and sphingolipids, was also generated.

## Materials and methods

### Production, purification and characterization of human wild-type recombinant α-synuclein

*Escherichia coli* BL21 (DE3) (Thermo Fisher, Waltham, MA) cells were transformed with a pET21a plasmid (courtesy of Prof. Outeiro's laboratory) for α-synuclein overexpression. After thawing, competent cells were incubated with the plasmid, heat-shocked at 42°C, and incubated in Super Optimal broth with Catabolite repression media at 37°C for 1 h. Cells were plated on Luria-Bertani (LB) agar with ampicillin (100 μg/mL) and incubated at 37°C overnight. A single colony was picked, grown in LB media supplemented with 100 μg/mL ampicillin and incubated overnight at 37°C with shaking at 200 rpm, and scaled up to 750 mL in the same conditions. The culture was placed in the shaking incubator until OD_600_ 0.6 was reached; at this point, 1 mM of IPTG was added, inducing protein production for 2 h. Cells were harvested through centrifugation at 4000 × g, producing a cell pellet. The cell pellet was resuspended in 30 mL of lysis buffer (750 mM NaCl, 10 mM Tris, pH 7.6, 1 mM EDTA) containing cOmplete™ mini protease cocktail inhibitor (Roche, Mannheim, GE) and ultrasonicated at 60% power, 30 s on and 30 s off for 5 min. The lysate was boiled, denaturing structured proteins that were removed via centrifugation, leaving α-synuclein in the supernatant. The sample was filtered via a 0.22 μm filter to remove contaminants and stored at −20°C. The ÄKTA FPLC system (Cytiva Life Sciences, Marlborough, MA) with a 1 mL Q HP HiTrap anion exchange column was primed and equilibrated with 5 column volumes (CV) of wash buffer (25 mM Tris, pH 7.6). The protein sample (500 μL) was injected, and the system washed with 5 CV of wash buffer. A linear gradient of elution buffer (1 M NaCl, 25 mM Tris, pH 7.6) up to 70% was used to elute proteins, with α-synuclein eluting at ∼60:40 elution/wash buffer. A final step to 100% elution buffer for 3 CV removed strongly bound proteins. Fractions containing α-synuclein, confirmed via silver staining, were concentrated using a 3 kDa MWCO filter. Further purification was performed using a Superdex 75 size-exclusion column (Cytiva Life Sciences, Marlborough, MA), equilibrated with running buffer (25 mM Tris, 100 mM NaCl, pH 7.6). The sample was injected, and a constant flow of elution buffer (0.8 mL/min) allowed fractionation. Positive fractions were concentrated to 5 mg/mL and stored in liquid nitrogen for future use. Purification and fibrillation processes were monitored using SDS-PAGE-silver staining, light scattering and LC-MS/MS for detailed protein analysis. α-Synuclein fibrils were assessed, and Raman analysis was performed during fibrillation, as described elsewhere.^41^

### Cell culture, transfection and PFF treatment

HEK293, Neuro2a and SH-SY5Y cells (Sigma-Aldrich; ^©^Merck KGaA, Darmstadt, Germany) were grown according to standard protocols in Gibco™ Dulbecco's Modified Eagle Medium (Gibco/Thermo Fisher Scientific; MA, USA), with 10% Gibco™ foetal bovine serum, and 1% Gibco™ antibiotic-antimycotic. In each experiment, three cell conditions were compared: untreated cells (neither transfected, nor treated with PFFs); cells transfected with a pcDNA3.1 plasmid (courtesy of Prof. Outeiro's lab) for α-synuclein overexpression; and treated cells, which were transfected and subsequently exposed to sonicated PFFs. Cells were grown until 70% confluence. For transfection and treatment, 2 μg of DNA and 3 μL of FuGENE® HD Transfection Reagent were mixed, incubated for 15 min, and 2 μL of the mixture was added to each well. Treated cells were left for 8 h before addition of sonicated PFFs. Briefly, aliquots of PFFs (0.1 mg/mL) were removed from −80°C storage and thawed at room temperature, before being sonicated at 60% for 1 min, 0.5 s on/off. Sonicated PFFs were added at a 1:100 concentration to media, which was added to pre-aspirated wells. Transfection and PFF addition occurred on the same day (0) with an 8-hour interval between. Cells were monitored for up to 14 days, with samples collected on Day 1, Day 7 and Day 14.

### Raman spectroscopy

Cells were grown for removal at Day 1, Day 7 and Day 14 in 12-well plates. At each timepoint the media was removed, the cells washed and removed by trypsinization for 3 min at 37°C. One hundred microliters of DMEM was added trypsinized cells, which were then gently resuspended. From this solution, 20 μL was removed and added to polished stainless-steel slides, pre-heated to 37°C. The solution was left for 5 min, after which deionized H_2_O was used to gently clean the slide, removing the media but leaving cells fixed to the slide. Cells were left to airdry for 5 min to ensure evaporation of water, leaving all the cells in a similar state before measurement. At each timepoint, 40 cells were analysed using an inVia™ confocal Raman microscope (Renishaw, Gloucestershire, UK), with a 785 nm laser at 100% power (∼110 mW) under a ×50 objective. A static scan was performed using a 1200 I/mm (633/780 nm) grating. Each measurement lasted 10 s, with six accumulations. To assess photodamaging of cells, a single cell was repeatedly assessed using the measurement above, over a 2 h period. Cellular spectral patterns were recorded and assessed, revealing a lack of spectral fluctuations over the 2 h period, suggesting that photodegradation was absent. Raman spectral data were pre-processed using WiRE software (Renishaw, Gloucestershire, UK) to perform a spline baseline subtraction of order 17. All spectra were recorded between 400 and 1800 cm^−1^ wavenumber range (1 cm^−1^ spectral resolution).

### Generation of an in-house lipid standards database

A Raman spectra library was created from spectral acquisition of lipid standards comprising sphingomyelin (Sigma Aldrich, 85615, from chicken egg yolk, 98%), galactocerebrosides (Sigma Aldrich, C4905, from bovine brain, 97%), ganglioside GM1 (Sigma Aldrich, 860065P, from ovine brain, 99%), L-α-phosphatidylinositol sodium salt (Sigma Aldrich, P0639, from Glycine max soybean, 99%), cerebrosides (Sigma Aldrich, 131303P, from ovine and ovine brain, 99%) and 1,2-dipalmitoyl-sn-glycero-3-phosphoethanolamine (Sigma Aldrich, P1348, 97%). Lipids were measured in powdered form on a stainless-steel slide. Measurements were taken with an inVia™ confocal Raman microscope. All spectra were recorded between 400 and 1800 cm^−1^ wavenumber range (1 cm^−1^ spectral resolution) with a 50× objective and a 785 nm (near infra-red) laser. For each spectral standard, five measurements were taken and averaged, with each measurement comprising an acquisition time of 10 s and six accumulations, performed at 100% (approximately, 110 mW) laser power at the sample surface. The baseline was automatically selected and subtracted, using a spline order of 13, and cosmic rays were removed with WiRE software (Renishaw, Gloucestershire, UK). Automated internal Raman calibration using the 520 cm^−1^ peak of a silicon wafer was performed before sample spectrum acquisition. Only the highest peaks (reaching at least 1000 intensity counts) were selected for inclusion in the database.

### Machine learning pipeline and statistical analysis

Spectral data were pre-processed in WiRE software, sorted in Microsoft Excel (Microsoft, Redmond, WA), and then imported into RStudio (©2024 Posit Software, PBC formerly RStudio, PBC), where the data underwent standard normal variate (SNV)-normalization,^42,43^ before undergoing a machine learning-based analysis. Firstly, principal component analysis (PCA) was applied to generate principal components (PCs) and identify the top PCs that explained the largest variations within the data. A subjective threshold of 70% of cumulative explained variance was applied to establish the number of PC to analyse in the loading plots, without investigating PCs, which may only explain low amounts of variance. Using a subjective threshold of ±0.075, influential peaks were established from loading plots, allowing identification of the wavenumbers that most contributed to variation within the dataset. Along with loading plot analysis, which identifies peaks of interest in an automated and unbiased manner, the Raman data also underwent visual analysis in the form of an overlay plot for each cell line and timepoint. Using both methods of analyses in tandem ensured that the peaks with the most variability were identified throughout the assay. Uniform manifold approximation and projection (UMAP) was also used to reduce the dimensionality of Raman spectroscopy data, allowing for the visualization of complex patterns and relationships between treated and untreated cells. Duplicates were removed, and each wavenumber was tested for significance, after establishing data normality through a Shapiro–Wilk test. Wavenumbers that were normally distributed were further analysed by ANOVA and then Tukey's Honestly Significance Difference *post hoc* testing, whereas non-normally distributed data were analysed via Kruskal–Wallis and Dunn–Bonferroni *post hoc* testing. The machine learning pipeline used can be found here: https://github.com/NColes2812/RamanSpectralAnalysis.

### Transmission electron microscopy

Untreated and treated cells from Day 14 were fixed, embedded and sectioned. Briefly, cells were pelleted at 3000 rpm for 15 min in a 1.5 mL Eppendorf tube, then turned 180° and re-spun at 3000 rpm for 15 min to create a flat cell pellet. The supernatant was removed and the cells resuspended in 2% glutaraldehyde in 0.1 M sodium cacodylate fixative, before being centrifuged again. The glutaraldehyde then removed and fresh fixative added. The samples were refrigerated until secondary fixation and agarose enrobing. The fixative was removed, and cells washed with sodium cacodylate buffer, then centrifuged. The buffer was removed, and the cells washed another two times before being resuspended in 1% osmium tetroxide, in sodium cacodylate and fixed for 1 h at 4°C. The osmium tetroxide was removed and sodium cacodylate used to wash the cells three times. Liquid agarose was added and the cells centrifuged at 4200 rpm for 1 min, then turned 180° and re-centrifuged. The cells were refrigerated for 30 min and the agarose pellet removed with a needle. The agarose was then cut into sections and dehydrated using acetone with increasing concentrations through 25%, 50% and 75% for 20 min and 100% acetone for 60 min, twice. Samples were then infiltrated with epoxy resin at 25%, 50% and 75% concentrations in acetone, each for 60 min then 100% resin in acetone, with buffers changed three times over 24 h. Finally, samples were embedded in 100% resin at 60°C for 1 day. Thin 0.5 μm sections were cut and stained with 1% toluidine blue in 1% borax. Using a diamond knife and ultramicrotome, 70 nm sections were cut and stretched with chloroform and mounted on a copper grid. Grids were stained with 2% aqueous uranyl acetate and lead citrate, and then captured using a Hitachi HT7800 TEM with a Emsis Xarosa camera with Radius software (EMSIS, GmbH).

### Immunofluorescence

Cells were grown in 12-well plates, each containing a Corning® BioCoat® poly-D-lysine coverslip (Corning, New York, USA) at the base of the well. Cells were cultured on the coverslip for up to 14 days, with coverslips removed on Day 1, Day 7 and Day 14 for assessment. Media was aspirated and the cells carefully washed twice with Dulbecco's Phosphate-Buffered Saline (dPBS), added drop-by-drop to ensure the cells remained adhered to the coverslips. Cells were then fixed in 4% paraformaldehyde for 15 min, at room temperature. After, the fixative was removed, the cells were carefully washed twice with dPBS, then incubated with 0.1% Triton X-100 in dPBS for 10 min. The cells were again washed in dPBS, and then blocked with 3% Bovine Serum Albumin in dPBS for 2 h. Following this, coverslips were incubated with antibodies for α-synuclein detection in BSA overnight at 4°C. The following primary antibodies were used: phospho-α-synuclein (Ser129) recombinant rabbit monoclonal antibody (JB22-44, Thermo Fisher Scientific, 1:200), and anti-aggregated α-synuclein antibody mouse clone 5G4 (MABN389, Sigma Aldrich, 1:1000). Cells were then gently washed twice with dPBS and incubated with secondary antibody in 1% BSA, for 2 h at room temperature. The following secondary antibodies were used: goat anti-rabbit IgG H&L Alexa Fluor® 647 (ab150083, Abcam, 1:1000) and goat anti-mouse IgG H&L Alexa Fluor® 488 (ab150114, Abcam, 1:1000). Lastly, the cells were gently washed twice with dPBS and fixed with FluoroShield™ mounting media with DAPI (Abcam, Cambridge, UK). Images from each timepoint and treatment method were taken on a Leica DMi8 inverted confocal microscope (Leica, Wetzlar, Germany).

### Cell viability assay

Cell viability assay was conducted on untreated and treated cells from the three cell lines, using the modified MTT assay kit protocol (Abcam, Cambridge, UK). Cells were seeded at a density of 10^4^ per well in 100 μL of growth media, in 96-well plates, and grown for 14 days, with media added every 2–3 days to avoid evaporation. On Day 1, Day 7 and Day 14, media was carefully aspirated from the wells, and they were washed briefly with dPBS, making sure as to not disturb the cells. Fifty microliters of dPBS and 50 μL of MTT reagent were added to the wells. The plate was incubated for 1 h at 37°C, after which 150 μL of MTT solvent was added. The plate was loaded into a BioTek Epoch™ 2 microplate reader (Agilent, CA, USA) and agitated for 15 min at 50 rpm, then the absorbance at 590 nm read. To calculate cell viability, replicate readings were averaged and the background absorbance from growth medium removed, giving a corrected absorbance. The following equation was then used to determine the cytotoxicity levels:


%Cytotoxicity=(100×(Control–Sample))/Control.

## Results

### α-Synuclein aggregation induces biochemical changes in protein, lipid and DNA profiles across multiple cell lines

Raman spectroscopy was employed to track biomolecular changes following the pathological aggregation of α-synuclein in HEK293, Neuro2a and SH-SY5Y. Peaks identified through loading plots and visual analysis were used to highlight regions of variability during the aggregation assay. All significant results (*P* < 0.05) were compiled and analysed using pre-established Raman spectral libraries (Supplementary Table 1) and previous studies and summarized in Table 1. On Day 1, all cell lines showed an increase in spectral peaks at 918, 1045^42^ and 1250 cm⁻^1^,^43-46^ which are associated with changes to β-sheet secondary structures. In HEK293 and SH-SY5Y cells, there was a noticeable decrease at 1659 cm⁻^1^, an amide I protein band linked to α-helical structures,^46,47^ suggesting pathological aggregation of α-synuclein into β-sheet-rich fibrils, similar to the fibrillation process seen in purified α-synuclein.^41,48^ By Day 14, HEK293 cells showed increases at 1045 and 1250 cm⁻^1^, further indicating the formation of β-sheet structures.^42-46^ Both HEK293 and Neuro2a cells exhibited a decrease in the amide I peaks at 1659 cm⁻^1^ on Day 14, suggesting a loss of α-helical structures.^46,47^ Tyrosine residues often engage in protein–protein interactions and their exposure during aggregation,^49^ aligning with a pathological shift from α-helical to β-sheet conformations seen in α-synuclein fibrillation. Accordingly, in SH-SY5Y cells, an increase was observed at 850 cm⁻^1^ on both Days 1 and 7, indicating the presence of tyrosine.^45,46,50^ By Days 7 and 14, Neuro2a cells showed elevated peaks at 838 and 850 cm⁻^1^, further suggesting an increase in tyrosine levels.^45,51^ Raman analysis of the three cell lines across the 14 days also identified upregulation in peak intensities at 1005 cm^−1^, commonly denoting phenyl ring breathing in phenylalanine and a downregulation in peak intensity at 1002 cm^−1^, identified as vibrational stretches of C–C bonds in phenylalanine or potential decrease in the presence of reactive oxygen species.^52-54^ On Day 14, there was a decrease at 780 cm⁻^1^ in both HEK293 and Neuro2a cells, corresponding to DNA perturbations,^45,49,55^ suggesting alterations in cellular DNA structure during the aggregation process.

**Table 1:** Summary of wavenumbers identified through analysis of spectral overlays and machine learning analysis of loading plots

Wavenumber (cm^−1^)	Allocation	D1 changes	D7 changes	D14 changes
724	Sphingomyelin	↓ HEK293, Neuro2a, SH-SY5Y	↓ Neuro2a, SH-SY5Y	↓ HEK293, Neuro2a, SH-SY5Y
780	DNA			↓ Neuro2a, SH-SY5Y
838	Tyrosine (ring breathing)			↑ HEK293, SH-SY5Y
850	Tyrosine	↑ SH-SY5Y	↑ Neuro2a, SH-SY5Y	↑ SH-SY5Y
918	Secondary structures	↑ HEK293, Neuro2a, SH-SY5Y		↓ HEK293
1002	Phenylalanine	↓ HEK293, Neuro2a, SH-SY5Y	↓ HEK293, Neuro2a, SH-SY5Y	↓ Neuro2a, SH-SY5Y
1005	Phenylalanine	↑ HEK293, Neuro2a, SH-SY5Y	↑ HEK293, Neuro2a, SH-SY5Y	↑ Neuro2a, SH-SY5Y
1045	β-Sheet formation	↑ HEK293, Neuro2a, SH-SY5Y		↑ HEK293
1060	Skeletal C–C stretch of lipids	↑ HEK293, SH-SY5Y		↑ HEK293
1125	Acyl backbone in lipids	↑ HEK293, Neuro2a, SH-SY5Y	↑ HEK293, ↓ Neuro2a, SH-SY5Y	↑ HEK293
1160	Conjugated v=C=C lipids	↑ HEK293, Neuro2a, SH-SY5Y	↓ Neuro2a, SH-SY5Y	↑ Neuro2a, ↓ SH-SY5Y
1315	CH3CH2 twisting mode of lipids	↓ HEK293, SH-SY5Y		↓ Neuro2a
1435	Lipids (CH3, CH2 deformations)	↓ Neuro2a, SH-SY5Y	↓ HEK293, Neuro2a, SH-SY5Y	↓ Neuro2a, SH-SY5Y
1659	Amide I	↓ HEK293, SH-SY5Y		↓ HEK293, SH-SY5Y

Each wavenumber showed a statistically significant increase (↑) or decrease (↓) in spectral intensity in treated cells compared to control cells.

Lipid changes were assessed using an in-house neurolipid library (Supplementary Table 1). This consisted of spectral standards from six common neurolipids, allowing identification of key lipid peaks, which were used to assess Raman cellular spectra at each stage of the 14-day assay. Through this analysis, lipid changes were apparent throughout the 14-day time course. On Day 1, HEK293 and SH-SY5Y cells showed an increase at 1060 cm⁻^1^, a peak corresponding to C–C stretches in lipids.^46,49,50^ Similarly, peaks at 1125 cm⁻^1^, associated with lipid acyl backbones,^49-51^ were elevated in all cell lines, while HEK293 cells exhibited an increase at 1160 cm⁻^1^, indicating the presence of gangliosides.^45^ However, lipid signals also showed decreases on Day 1, particularly in HEK293 and SH-SY5Y cells at 1315 cm⁻^1^, related to CH₃CH₂ twisting,^51^ and in Neuro2a and SH-SY5Y cells at 1435 cm⁻^1^, linked to CH₃ and CH₂ deformations in lipids.^51,56-59^ On Day 7, decreases were seen in lipid peaks at 1125 cm⁻^1^ in Neuro2a and SH-SY5Y cells, and at 1160 and 1435 cm⁻^1^ in HEK293 and Neuro2a cells.^16,51,52,56-61^

By Day 14, HEK293 cells demonstrated increases at 1060, 1125 and 1160 cm⁻^1^, further indicating lipid alterations.^45,46,49-51^ However, there were also decreases in lipid-related peaks across all cell lines at 1435 cm⁻^1^, corresponding to CH₃ and CH₂ lipid deformations,^44,49,55,58,61^ and a drop at 1315 cm⁻^1^ in HEK293 and SH-SY5Y cells, linked to CH₃CH₂ twisting of lipids.^51^ Furthermore, there was significant decreases in nearly all cell lines and timepoints at 724 cm^−1^, relating to the presence of sphingomyelin, suggesting a loss of this lipid or its involvement in molecular interactions.

**Figure 1:**
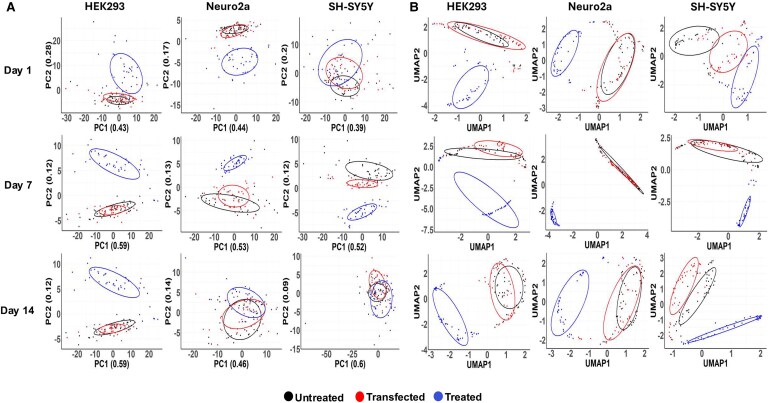
**PCA (A) and UMAP (B) plots showing group clustering of HEK293, Neuro2a and SH-SY5Y cells at different timepoints in the *in vitro* aggregation assay.** Forty spectra were acquired per cell line and plotted as dimension-reduced points for each treatment and cell line. (**A**) PCA plots show relative principal component (PC) values on corresponding axis, denoting weighted variance of each PC used to delineate treatment groups. (**B**) UMAP plots show which UMAP's were used to delineate treatment groups.

PCA was used to generate a PCA plot, mapping PC1 and PC2 for each cell line and timepoint to assess whether the spectral profiles of untreated, transfected and PFF-treated cells were different (Fig. 1A). On Day 1 of the assay, treated HEK293 and Neuro2a cells clustered distinctly from untreated and transfected-only cells, indicating significant spectral changes between these groups. SH-SY5Y treated cells on Day 1 shared a large overlap with transfected cells and a small overlap with untreated cells, suggesting a more gradual cellular response to α-synuclein overexpression and aggregation. On Day 7 of the assay, treated cells distinctly grouped apart from untreated and transfected cells across all cell lines, suggesting that PFF treatment induced significant cellular biochemical changes. On Day 14, overlap was seen between treated and transfected cell lines, potentially indicating proliferation of new cells or clearance mechanisms removing the protein upon pathological aggregation. Alongside PCA, the SNV-normalized Raman data were analysed via UMAP analysis for dimension reduction and classification. Ten seeds were used, and the results averaged, providing a stable and robust analysis method. UMAP analysis accurately differentiated between treated and transfected/untreated cells at each stage of the assay, in each cell line (Fig. 1B). On Day 1, untreated and transfection-only HEK293 and Neuro2a cells showed distinct overlap, whereas in SH-SY5Y cells these groups showed distinct grouping, suggesting that the overexpression of α-synuclein alone also caused distinct cellular changes. On Day 7 and Day 14, all cell lines grouped distinctly, with a large degree of delineation between treated, and untreated and transfection-only cells, mirroring the substantial spectral changes occurring in these cells.

### Cellular effects of α-synuclein expression and PFF treatment

**Figure 2:**
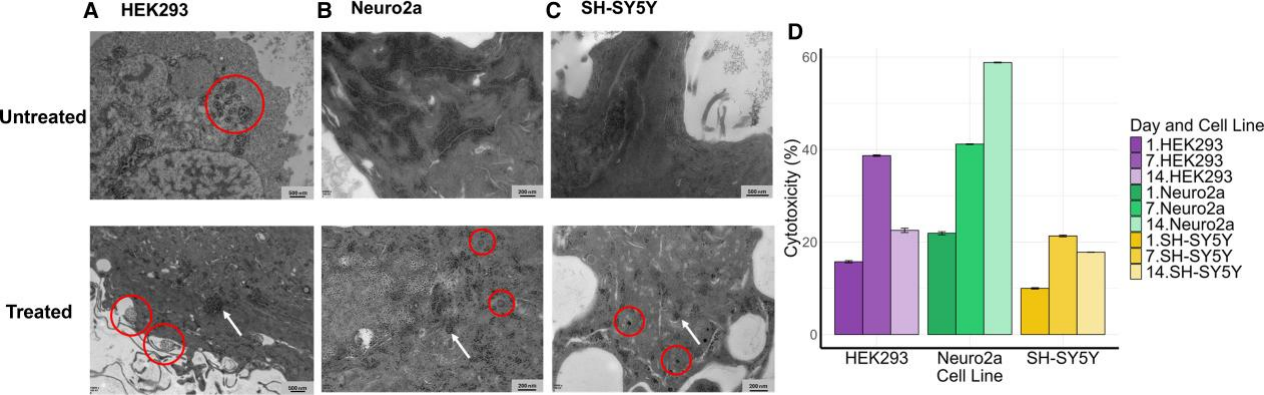
**TEM images and cell viability assay of cell lines before and after pre-formed fibril treatment (at Day 14).** (**A**) HEK293 cells: untreated HEK293 cells with visible mitochondria (circle). After transfection and treatment, larger dark intracellular aggregates become visible (arrow). Additionally, distinct cellular projections are seen, potentially indicating exocytosis (circle). (**B**) TEM images of Neuro2a cells: untreated Neuro2a cells with dark regions of dense aggregates. After transfection and treatment, small dense aggregates and larger granular aggregates, similar to those seen in HEK293 cells (arrow), are visible. Small spherical structures (circle) are also apparent. (**C**) TEM images of SH-SY5Y cells: untreated SH-SY5Y cells with dark regions comprised of small dense aggregates. After transfection and treatment, SH-SY5Y cells exhibit small dense aggregates alongside larger granular aggregates, resembling those in HEK293 and Neuro2a cells (arrow). Larger aggregates are also visible in treated cells (circle). (**D**) Cell viability assay: cytotoxic effects of α-synuclein overexpression and treatment with sonicated PFFs with cell viability assay. Using untreated cells as the baseline, HEK293 and SH-SY5Y cells show a slight increase in cytotoxicity from Day 1 to Day 7, followed by a decrease by Day 14. Neuro2a cells exhibit a gradual increase in cytotoxicity over 14 days. Data are presented as mean ± SD of triplicate technical replicates, expressed as mean ± SD, and shown as a percentage of cytotoxicity.

Using thin section TEM, untreated HEK293 cells displayed clear cellular features, including distinct mitochondria and a spheroidal structure. (Figure 2A) In contrast, after treatment with sonicated PFFs, the cells exhibited large, dark granular inclusions distributed throughout the cytoplasm (Fig. 2A; white arrow). Additionally, the cells developed projections, indicating possible vesicular exocytosis of cellular material (Fig. 2A; red circles). Untreated Neuro2a cells exhibited small, dense aggregates, while treated Neuro2a cells showed small dense aggregates, and larger granular aggregates, similar to those observed in HEK293 cells (Fig. 2B, white arrow). Treated Neuro2a cells also showed bilayered vesicles (Fig. 2B, red circles). Untreated SH-SY5Y cells displayed dark, dense aggregates dispersed throughout the cells, which appeared visually distinct from the larger granular aggregates observed in treated HEK293 and Neuro2a cells (Fig. 2C). Treated SH-SY5Y cells showed larger aggregates, potentially indicating the formation of proteinaceous inclusion bodies (Fig. 2C; red circles). A cell viability assay was also conducted to assess the impact of α-synuclein PFF treatment on cells overexpressing α-synuclein, focusing on cellular viability. In Neuro2a cells, increases in cytotoxicity were observed from Day 1 to Day 7 (*P* = 0.06), and to Day 14. In HEK293 and SH-SY5Y cells, cytotoxicity increased from Day 1 to Day 7 but decreased from Day 7 to Day 14 (Fig. 2D). This reduction in cytotoxicity suggests that clearance mechanisms may be reducing cytotoxic effects in the remaining viable cells over time.

**Figure 3:**
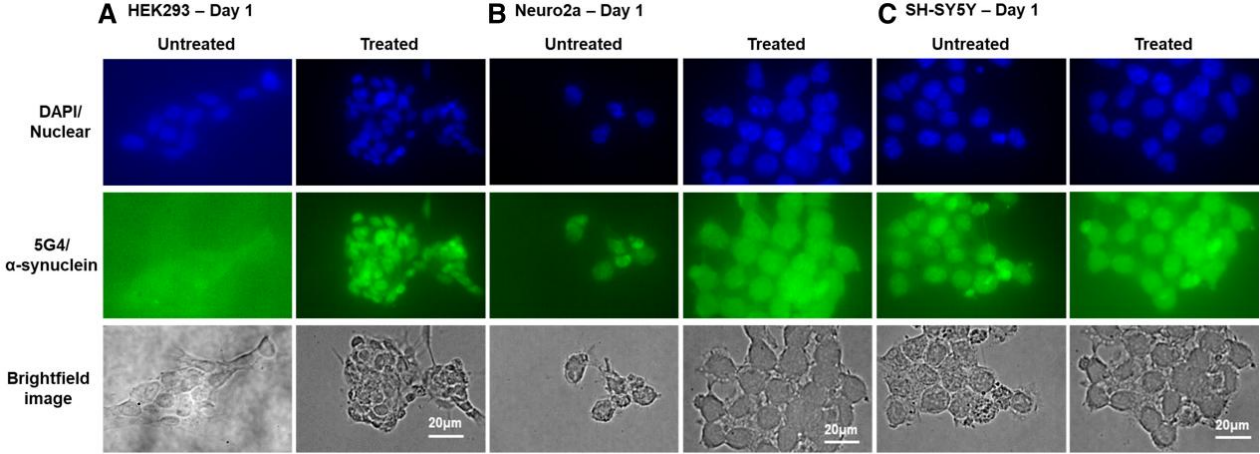
**Immunofluorescent staining of HEK293 (A), Neuro2a (B) and SH-SY5Y (C) cells at Day 1.** (**A**) Untreated HEK293 cells showed no detectable α-synuclein (5G4) expression. In contrast, treated cells exhibited higher levels of α-synuclein expression compared to untreated cells, validating plasmid efficacy. (**B**) Untreated Neuro2a cells show aggregated α-synuclein (5G4); however, treated cells exhibited higher levels of α-synuclein expression compared to untreated cells on Day 1. (**C**) Untreated SH-SY5Y cells show expression of aggregated α-synuclein (5G4) with expression of α-synuclein qualitatively higher in treated cells on Day 1.

To assess the efficacy of plasmid-based α-synuclein expression and establish qualitative levels of α-synuclein, immunofluorescence assays were conducted on HEK293, Neuro2a and SH-SY5Y cells at each stage of the aggregation assay (Fig. 3). As expected, α-synuclein was not expressed in untreated HEK293 cells (Fig. 3A). After transfection, α-synuclein expression was detected in HEK293 cells using the 5G4 antibody on Day 1 (Fig. 3A). Overall, total 5G4-GFP expression increased in cells treated with PFFs compared to those transfected with the plasmid alone (Fig. 3A). Untreated Neuro2a cells showed moderate levels of 5G4 α-synuclein expression on Day 1, with transfection not affecting overall expression levels. Following PFF treatment on Day 1, an increased number of Neuro2a cells showed elevated 5G4 α-synuclein expression, although the intensity and amount per cell remained unchanged. (Figure 3B). For SH-SY5Y cells, on Day 1, untreated cells showed moderate levels of 5G4 α-synuclein expression, with transfection alone not affecting these levels. After treatment with PFFs on Day 1, SH-SY5Y cells exhibited increased expression of 5G4 α-synuclein (Fig. 3C).

## Discussion

This study aimed to assess the cellular impact of α-synuclein aggregation upon seeding with sonicated PFFs. Raman spectroscopy was employed to monitor the effects of aggregation over a 14-day period, with key peaks and patterns analysed through a machine learning pipeline. Changes in Raman spectral peak intensity provided insights into dynamic molecular changes within mammalian cells, including early α-synuclein aggregation stages and key changes in cellular makeup during early nucleation. Significant increases in peaks relating to β-sheet formation after the transfection and PFF treatment of three mammalian cells lines, suggesting rapid fibrillation. Furthermore, distinct changes in lipid profiles of treated mammalian cells were found, with peaks relating to lipids, notably sphingomyelin, showing decreases in intensities over the 14-day *in vitro* aggregation assay.

Distinct signs of protein aggregation were identified on Day 1 for all three treated cell lines. Increases were seen at 918 cm^−1^ (secondary structures), 1045 cm^−1^ (β-sheet formation) and 1250 cm^−1^ (amide III/β-sheet formation), while decreases were seen at 1659 cm^−1^ (amide I/α-helical structures). Taken together, this suggests that α-synuclein rapidly aggregated into β-sheet-rich fibrillar structures, as found during the fibrillation process of the purified α-synuclein used to create PFFs for this study.^41^ These peaks were not observed after Day 1, suggesting that aggregates might be removed from the cell via exocytosis.^40^ Alongside the observed changes in α-synuclein conformation, Raman analysis of the three cell lines over 14 days revealed significant alterations in amino acid environmental profiles. Specifically, there was an upregulation in peak intensity at 1005 cm⁻^1^, associated with phenyl ring breathing in phenylalanine and a downregulation at 1001 cm⁻^1^, linked to vibrational stretches of C–C bonds in phenylalanine.^52,53^ Under oxidative stress conditions, such as those produced by the presence of HMW α-synuclein oligomers,^18^ carbon three of the phenyl ring of phenylalanine can become oxidized by a hydroxyl radical, leading to the production of *m-*tyrosine^60^ This binding may affect the intramolecular conformation of the phenyl-ring, reducing intensities at 1001 cm^−1^, and increasing intensities of the 1005 cm^−1^ peak, as reported here. Sporadic increases in intensities were also seen at 830 and 850 cm^−1^, on Day 7 and Day 14 across the cell lines, further corroborating the conversion of phenylalanine to *m*-tyrosine.^57^ This suggests that HMW α-synuclein oligomers trigger oxidative stress conditions and conversion of phenylalanine to *m-*tyrosine. Furthermore, oxidative stress was also suggested via visual analysis of TEM images in the form of abundant spherical double-ringed organelles, visibly like autophagosomes, in treated Neuro2a cells (Fig. 2B), vacuolation in SH-SY5Y cells (Fig. 2C). Autophagosomes break down cellular components due to oxidative stress^59^ and are thought to play a central role in the disruption of the autophagy-lysosomal pathway associated with α-synuclein aggregation. Altered levels of autophagy markers have been observed in the brains of individuals with Parkinson's disease,^56,62,63^ DLB^63^ and multiple systems atrophy (MSA).^64^ This suggests that pathological α-synuclein aggregates may induce oxidative stress, leading to increased autophagosomes that attempt to clear aggregation by-products such as the pathological β-sheet structures of α-synuclein, potentially explaining the reduction in β-sheet peaks after Day 1 in this experiment. Prolonged autophagosomal activity may therefore disrupt cellular systems, allowing unchecked α-synuclein aggregation to persist. Such increased autophagosomal activity may also be supported by the increase of ceramide signal detected following treatment. Neuro2a cells displayed a high number of bilayered vesicles, potentially autophagosomes, where sphingomyelin is converted to ceramide by acidic sphingomyelinase.^65^ The increased presence of these vesicles could thus explain the upregulation of ceramide found during the aggregation process.^66^ Elevated ceramide levels have also been reported in the plasma of DLB patients.^22,67,68^ In multiple systems atrophy, which is characterized by α-synuclein deposits in oligodendrocytes, decreased sphingomyelin levels negatively correlated with α-synuclein expression.^69^

**Figure 4:**
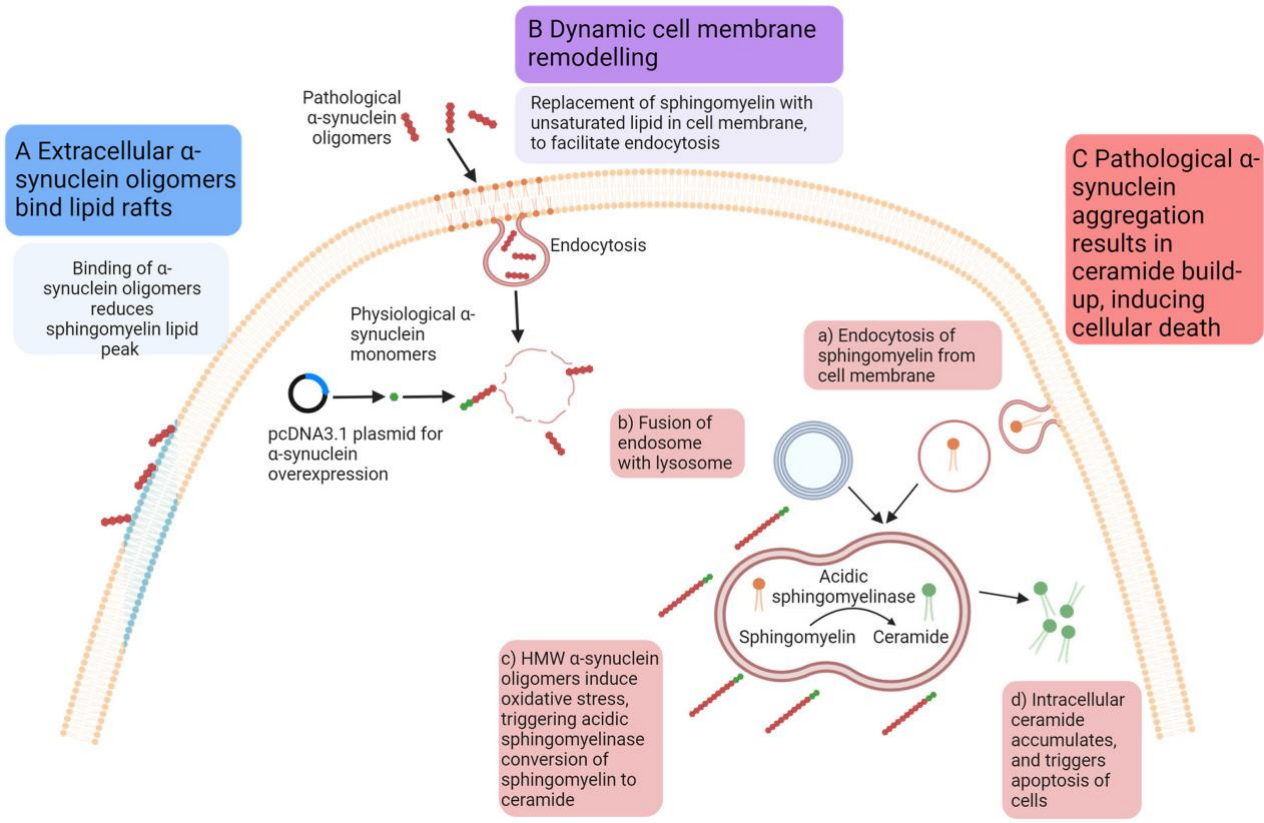
**Three hypothesized mechanisms explaining the observed decrease in sphingomyelin peaks.** (**A**) The ability of α-synuclein oligomers to bind lipid rafts, potentially altering intramolecular bonding and affecting the Raman signal. (**B**) Cellular membrane remodelling through lipid restructuring, which facilitates the endocytosis of extracellular proteins like α-synuclein oligomers. (**C**) Pathological α-synuclein oligomers induce lysosomal stress, leading to the conversion of sphingomyelin to ceramide, which in turn triggers apoptosis. This mechanism accounts for the loss of sphingomyelin peaks, the increase in ceramide acyl backbone peaks and the decrease in DNA peaks observed over the 14-day *in vitro* aggregation period (created in BioRender. Butler, L. 2025 https://BioRender.com/g63e507).

On PFF treatment, TEM analysis revealed small spherical aggregates in all cell lines (Fig. 2), which were absent in untreated cells. Initially, HEK293 cells showed no α-synuclein expression via immunofluorescence (Fig. 3), corresponding to the absence of aggregates under TEM. In contrast, Neuro2a and SH-SY5Y cells expressed α-synuclein without visible aggregates. After treatment, immunofluorescence staining indicated α-synuclein expression in all cell lines, along with aggregate formation. This suggests that the aggregates observed are not solely composed of α-synuclein but may also include by-products of α-synuclein-mediated damage. Exocytosis in treated HEK293 cells may indicate the cell's attempt to expel these by-products rather than α-synuclein aggregates. A significant decrease at 724 cm⁻^1^, a lipid peak related to sphingomyelin^46,50,70^ (Table 1) on Day 1 was found in all cell lines. Although the cause of this decrease is unclear, it could be explained by several mechanisms (Fig. 4). First, extracellular α-synuclein oligomers may bind to lipid rafts, which are cholesterol- and sphingomyelin-rich membrane domains involved in membrane fluidity and signalling.^71^ As α-synuclein specifically associates with lipid rafts, it may affect various signalling pathways linked to cellular death.^72,73^ For instance, the A30P mutation linked to Parkinson's disease disrupts α-synuclein interaction with lipid rafts,^72^ a critical process for its synaptic localization. This highlights the crucial role of lipid rafts in α-synuclein normal function, and perturbations in this association may drive changes in α-synuclein contributing to Parkinson's disease pathogenesis.^72^ Furthermore, this binding could disrupt sphingomyelin bond vibrations, reducing intensities at 724 cm⁻^1^. Another explanation is that the decrease in intensity at 724 cm⁻^1^ may reflect a loss of sphingomyelin from the cell membrane. Sphingomyelin is supported by a rigid membrane, and its loss could increase the ratio of unsaturated fatty acids, making the membrane more dynamic and facilitating endocytosis.^74,75^ This flexibility could promote the uptake of oligomeric α-synuclein from the extracellular environment. Supporting this idea, HEK293 cells exhibited increases in peaks related to C–C skeletal stretching (1060 cm⁻^1^), the lipid acyl backbone (1125 cm⁻^1^) and unsaturated C=C bonding (1160 cm⁻^1^), indicating upregulation of unsaturated lipids. Another possibility is that α-synuclein aggregation reduces sphingomyelin levels by promoting its conversion into ceramide or inhibiting its reconversion to sphingomyelin.^76^ HMW α-synuclein oligomers induce oxidative stress, triggering lysosomal conversion of sphingomyelin to ceramide via acidic sphingomyelinase.^18^ Ceramide, which has a distinct Raman peak at 1127 cm⁻^1^, was found to be upregulated in this study, suggesting that sphingomyelin-to-ceramide conversion could be tracked through changes in Raman spectra. TEM examination identified small intracellular inclusions in all treated cell lines, also observed undergoing exocytosis in HEK293 cells (Fig. 2A; red circles). These inclusions, likely containing not only α-synuclein but also ceramide, suggest early formations of Lewy bodies. α-Synuclein may bind to intracellular lipids, facilitating the formation of aggregates.^20,77^

The build-up of ceramide within mammalian cells has been linked to cell death in several studies.^78-80^ A recent study found that HMW α-synuclein oligomers directly induce ceramide production via sphingomyelinase, leading to caspase-independent apoptosis.^18^ On Day 14 of the assay, treated HEK293 and Neuro2a cells showed significant decreases at 780 cm⁻^1^, a peak associated with DNA. This peak has been reported to decrease during apoptosis in cancer cells,^38^ further suggesting apoptotic cell death in HEK293 and Neuro2a cells. Apoptosis also corresponded with decreases in protein and lipid peaks, likely due to the fragmentation of cellular components.^38^ On Day 14, decreases in general lipid and protein peaks were observed, specifically at 1315 cm⁻^1^ (associated with CH3CH2 twisting in lipids), 1435 cm⁻^1^ (relating to CH3 and CH2 deformations) and 1659 cm⁻^1^ (an amide I peak representing proteins) in HEK293 and Neuro2a cells, suggesting apoptotic cell death and nuclear fragmentation. The MTT cell viability assay corroborated these findings, showing non-significant increases in cytotoxicity in HEK293 and SH-SY5Y cells until Day 7, with a similar increase noted in Neuro2a cells by Day 14.

Raman spectroscopy, combined with a machine learning, was used to assess dynamic biomolecular changes during pathological α-synuclein aggregation. PCA and UMAP analyses were employed to differentiate between treated and untreated cells at each timepoint, assessing the impact of α-synuclein aggregation. PCA did not reveal any separation between treated and transfected cell lines (Fig. 1A). However, UMAP analysis showed that treated cells grouped distinctly from both untreated and transfected cells for each cell line and timepoint. A significant overlap between untreated and transfected cells was observed, suggesting that overexpression of α-synuclein alone did not sufficiently alter cellular dynamics (Fig. 1B). The clear separation of treated cells from transfected ones indicates that sonicated PFFs were effectively recruited into the cell, where they aggregated with the overexpressed α-synuclein. This aggregation sufficiently impacted cellular function and structure, enabling Raman spectroscopy to detect these distinctions.

While α-synuclein aggregation appears linked to ceramide production and apoptosis, autophagy and mitochondrial dysfunction were not explored. A more detailed investigation into these pathways could offer a deeper understanding of how lipid dysregulation contributes to cell death and neurotoxicity in this context. Although Day 14 samples allowed for observable α-synuclein-induced changes, potential cell proliferation may have confounded results. Measures were taken to mitigate this by removing non-adherent dead cells.

## Conclusion

In conclusion, distinct changes in protein aggregation were observed across all treated cell lines on Day 1 using Raman spectroscopy, marked by increased β-sheet formation (918 cm⁻^1^, 1045 cm⁻^1^, 1250 cm⁻^1^) and decreased α-helical structures (1659 cm⁻^1^), likely relating to structural changes in α-synuclein protein. These results indicate that α-synuclein rapidly aggregated into β-sheet-rich fibrils, consistent with the fibrillation process. Sporadic increases at 830 and 850 cm⁻^1^ on Day 7 and Day 14 suggest oxidative stress-induced conversion of phenylalanine to *m-*tyrosine. This is further supported by TEM imaging showing autophagosome-like structures and vacuolation in treated cells, linking α-synuclein aggregation to oxidative stress and cellular damage. Distinct alterations in lipid profiles were a feature in treated cells. A significant decrease in sphingomyelin-related lipid peaks (724 cm⁻^1^) on Day 1 was seen across all cell lines and timepoints, suggesting potential α-synuclein-induced disruptions from in lipid rafts, crucial for membrane fluidity and contributing to cellular dysfunction. The observed increase in unsaturated lipids, along with a rise in ceramide levels, suggested a sphingomyelin-to-ceramide conversion, likely triggered by oxidative stress, supported by TEM identification of intracellular inclusions and autophagosome-like structures, indicating early aggregate formation and lipid disruption. Decreases in lipid and protein peaks on Day 14, along with cell viability assay, indicated apoptotic cell death, with α-synuclein accumulation, playing a role in cytotoxicity. Consistent with recent studies, these results highlight the central role of disrupted lipid homeostasis in α-synuclein aggregation, driving neurotoxicity and cell death in synucleinopathies.

## Supplementary Material

fcaf133_Supplementary_Data

## Data Availability

The code used for Raman spectral analysis in this study is publicly available in the GitHub repository at the following URL: https://github.com/NColes2812/RamanSpectralAnalysis. The data generated and analysed during this study are available from the corresponding author upon reasonable request.
