# Supplementary material for: Alpha-synuclein aggregation induces prominent cellular lipid changes as revealed by Raman spectroscopy and machine learning analysis
Source: Brain Commun. 2025 Apr 3;7(2):fcaf133. doi: 10.1093/braincomms/fcaf133 (PMC11992568; doi:10.1093/braincomms/fcaf133)
Supplement: fcaf133_Supplementary_Data [file fcaf133_supplementary_data.docx]

**Supplementary Table 1.** Raman spectral peaks found within six lipids of the in-house library, with bold numbers identifying major spectral peaks.

| **Lipid** | **Wavenumbers (cm^-1^)** |
| --- | --- |
| **Galactocerebrosides** | 680, 870, 890, **1060**, 1080, 1110, **1125**, **1295**, 1370, **1430**, **1455**, 1555, **1655**, **1670** |
| **Gangliosides** | **670**, **680**, 830, **870**, 940, 975, **1060**, 1080, **1125**, 1155, **1295**, **1375**, **1430**, 1455, 1555, **1670** |
| **L-a-phosphatidylinositol sodium salt** | 680, 815, 845, 870, 890, 940, **1075**, 1120, **1300**, 1370, **1440**, 1555, **1635**, **1655**, 1735 |
| **Cerebrosides** | 700, 820, 870, **885**, 925, 945, 1030, **1060**, **1080**, **1110**, **1130**, 1175, 1250, **1290**, **1370**, **1435**, **1455**, 1540, **1625**, **1655**, **1675** |
| **Dipalmitoyl** | 845, 870, **885**, 990, **1060**, **1095**, **1125**, 1175, **1295**, 1370, **1430**, **1455**, 1730 |
| **Sphingomyelin** | 845, 870, **885**, 990, **1060**, **1095**, **1125**, 1175, **1295**, 1370, **1430**, **1455**, 1730 |
